# Personality Traits and Treatment Adherence Among Parents of Children with Atopic Dermatitis

**DOI:** 10.3390/medicina62010059

**Published:** 2025-12-28

**Authors:** Adela Markota Čagalj, Zdenka Šitum Čeprnja, Dina Lešin Gaćina, Jasna Petrić Duvnjak, Maja Pavić, Tina Gogić Salapić, Bepa Pavlić, Shelly Melissa Pranić, Dubravka Vuković

**Affiliations:** 1 Department of Dermatology and Venereology, University Hospital of Split, Spinčićeva 1, 21000 Split, Croatia; adela.markota@gmail.com (A.M.Č.); 2 Department of Ophthalmology, Zagreb University Hospital Center, Kišpatićeva 12, 10000 Zagreb, Croatia; 3 Poliklinika Pediatri, Kranjčevićeva 45, 21000 Split, Croatia; 4 Department of Health Studies, University of Split, Ulica Ruđera Boškovića 35, 21000 Split, Croatia; 5 Department of Public Health, School of Medicine, University of Split, Šoltanska 2, 21000 Split, Croatia; 6 Cohrane Croatia, Šoltanska 2, 21000 Split, Croatia; 7 School of Medicine, University of Split, Šoltanska 2, 21000 Split, Croatia

**Keywords:** atopic dermatitis, parents, personality traits, adherence

## Abstract

*Background and Objectives*: Atopic dermatitis (AD) is a prevalent, chronic, relapsing itchy skin disorder, affecting up to 20% of the pediatric population. Topical corticosteroids are the cornerstone of AD treatment, but their use is often limited due to topical corticosteroid phobia among parents. Research on chronic illnesses highlights the significant role of personality traits in treatment adherence, with emotional stability and conscientiousness—within the framework of the Five-Factor Model—emerging as key predictors. The aim of our study was to examine the relationship between parental personality traits and their adherence to the treatment of their children with AD. *Materials and Methods*: A cross-sectional study was conducted at the Department of Dermatovenereology, University Hospital of Split, involving 90 parents of children diagnosed with AD. Personality traits were evaluated using the abbreviated version of the International Personality Item Pool Big-Five Personality Questionnaire (IPIP 50s). Treatment adherence was assessed through a valid and reliable questionnaire, the Morisky Medication Adherence Scale (MMAS-8). Statistical analyses were performed using JASP v.0.18.1.0. *Results*: According to MMAS-8, only a small proportion of the sample reported having high adherence (14.4%). The only significant associations between personality traits and adherence were found between conscientiousness and adherence and emotional stability and adherence, where more conscientious participants and more emotionally stable participants reported higher scores. *Conclusions*: The results suggest that parents of children with AD with higher scores on conscientiousness and emotional stability are more likely to demonstrate better treatment adherence. These insights may encourage a holistic and multidisciplinary approach to the treatment of children with AD, with an emphasis on providing psychological support to both the children and their parents in order to improve treatment adherence and the further clinical course of the disease.

## 1. Introduction

Often starting in infancy or early childhood, atopic dermatitis (AD) is a common chronic inflammatory skin condition that has a relapsing–remitting pattern [1]. Intense itching, eczematous lesions, disturbed sleep, and significant detrimental impacts on children’s and their families’ health-related quality of life are its hallmarks [2]. According to recent epidemiological estimates, AD affects a significant portion of pediatric populations; estimates vary by area and age and are sometimes estimated as high as 15% to 20% in youngsters. [3]. Global Burden of Disease (GBD) 2019 data show that the prevalence of AD among children under 14 years in Croatia was 5.42% [4].

The primary approach to managing pediatric AD is skin-directed and multifaceted, involving frequent use of emollients, avoiding triggers, and gradually treating inflammation (usually with topical corticosteroids for flare-ups and topical calcineurin inhibitors or systemic therapies for specific cases), as well as patient/caregiver education and follow-up [5]. Caregiver behavior and adherence to prescribed regimens are key factors in determining disease control and clinical outcomes because parents and other caregivers are usually in charge of young children’s daily skin care and decision-making [6].

Adherence to suggested topical and supportive regimens in pediatric AD is typically inadequate, despite the availability of effective therapies [7]. Non-adherence is linked to more frequent flare-ups, higher symptom load, and higher healthcare utilization. It might manifest as improper dose, inconsistent or incorrect topical corticosteroid application, early withdrawal, or underuse of emollients [8]. Low real-world adherence to topical regimens in AD is described by observational and electronic-monitoring research; systematic reviews point to regimen complexity, caregiver knowledge gaps, and corticosteroid safety concerns as common causes of poor adherence [8].

Topical corticosteroid phobia, also known as corticophobia, is a prevalent and well-documented obstacle to effective topical steroid usage across caregiver groups. It consists of anxieties and beliefs regarding local and systemic adverse effects, skin thinning, dependence, and long-term harm [9]. Research indicates that higher levels of corticophobia in parents are associated with lower steroid use and less effective treatment implementation. Validated tools, such as the TOPICOP questionnaire, have been created to measure corticophobia [9,10]. Corticophobia is still prevalent and clinically significant among parents and caregivers of children with AD, according to recent studies [11,12].

In contrast to the extensive research on situational, regimen-related, and cognitive factors (such as disease knowledge, health literacy, and provider communication) as determinants of adherence, dermatology has paid relatively less attention to more stable dispositional factors, particularly caregiver personality [13,14]. The Five-Factor Model (FFM), which includes conscientiousness, emotional stability (neuroticism), extraversion, agreeableness, and openness, offers a condensed framework for conceptualizing individual differences that may influence behaviors related to health [15,16]. In more general health research, lower emotional stability is linked to emotional reactivity and variable associations with adherence (both risk and protective patterns have been observed depending on context), while higher conscientiousness is consistently linked to organized, planful behavior and better adherence to medical recommendations [13,14,17]. Additional FFM traits, including agreeableness, openness, and extraversion, may have an impact on seeking treatment, interacting with medical professionals, and being open to new management techniques [14].

Parents’ broader personality profiles may therefore influence not only the implementation of practical routines but also help-seeking and openness to education. Recent models of adherence emphasize that both cognitive beliefs regarding medicines and dispositional tendencies jointly influence treatment implementation. Consequently, the integration of personality with established cognitive frameworks, such as the Necessity–Concerns framework, can enhance predictions regarding which caregivers may encounter difficulties with topical regimens [18,19]. Interventions that effectively modify adherence in various chronic conditions frequently integrate belief-targeted education with behavioral supports (reminders, action plans) that address trait-related vulnerabilities (for example, low conscientiousness). This indicates a distinct translational pathway from personality assessment to customized adherence support in pediatric AD [20,21,22].

Empirical data suggest that personality traits matter for medication-taking behavior across many chronic illnesses, but studies explicitly investigating parental personality and treatment adherence in pediatric AD are scarce [13,14,17,23]. The clinical characteristics of AD, such as its need for topical treatments, sporadic flare-ups, and widespread corticophobia, provide an environment where parental dispositional tendencies may have very potent consequences [9]. For instance, higher parental conscientiousness may make it easier to establish and maintain regular emollient routines and the appropriate stepwise use of topical steroids. Lower parental emotional stability (higher neuroticism) can result in either excessive treatment (out of worry) or treatment avoidance (out of fear), depending on how worry and beliefs interact [17]. Additionally, personality may mitigate the effect of external stresses (such as caregiver strain and sleep disturbance) that are typical in families of children with AD on adherence [23].

Methodologically, important gaps limit our understanding of the role of dispositional traits in shaping caregivers’ adherence to prescribed regimens for children with AD. Adherence has been measured in different ways (self-report, prescription-refill records, electronic monitoring), which complicates cross-study comparisons. Many studies also do not adjust for key confounders such as disease severity, socioeconomic status, and health literacy, whereas dermatology-specific investigations of parental personality and adherence are limited [5,24,25]. Addressing these limitations matters for both theory and practice: incorporating dispositional predictors into adherence models complements existing situational and cognitive determinants and may help clinicians identify families at risk of poor treatment implementation [5,8,14].

By clarifying dispositional and cognitive contributors to parental treatment implementation in pediatric AD, this research aims to inform more personalized, psychologically informed adherence support within multidisciplinary atopy care—potentially improving treatment uptake, symptom control, and family quality of life. The findings may also stimulate further longitudinal and intervention research testing whether personality-tailored adherence support can produce meaningful clinical benefits in pediatric dermatology.

## 2. Materials and Methods

### 2.1. Study Design

From June 2024 to March 2025, we conducted a cross-sectional questionnaire study within the Department of Dermatology and Venereology, University Hospital of Split, enrolling parents of children who attended routine follow-up with a pediatric dermatologist for AD-related visits. Parents were invited to the clinic’s Atopy School, which was organized within the department, and during those sessions, consenting participants completed the study questionnaires using paper and pencil. The instruments used in this study were the abbreviated International Personality Item Pool 50-item questionnaire (IPIP-50) [15,16] to assess parental personality traits and the 8-item Morisky Medication Adherence Scale (MMAS-8) [26,27] to assess self-reported adherence to the child’s prescribed topical/supportive regimen. Participants were also asked to complete a supplementary demographic and clinical form. This study involved parents or legal guardians of children aged 0–14 years who had previously been diagnosed with AD by a dermatologist according to standard clinical criteria (Hanifin and Rajka) [28]. Eligible participants were parents/legal guardians who attended the clinic’s Atopy School, were able to provide written informed consent, and had sufficient Croatian language proficiency to complete the questionnaires. Children had to have received topical corticosteroid therapy at least once during the three months preceding enrollment. Exclusion criteria applied to parents/legal guardians of children with acute non-dermatologic illnesses that precluded clinic attendance or whose child was receiving systemic immunosuppressive treatment for indications unrelated to AD. The study protocol was approved by the Ethics Committee of the University Hospital of Split (520-03/24-01/143, 27 June 2024) and by the Ethics Committee of the School of Medicine, University of Split (029-01/24-02-0001, 23 July 2024).

### 2.2. Demographic Information

Participants completed a demographic and clinical form that collected parental age, sex, marital status, highest education level, employment status, number of children, tobacco use, and whether the respondent worked in healthcare. The form also recorded health-related variables, including chronic comorbidities, a personal or family history of atopic disease (AD, asthma, allergic rhinitis), and prior or current use of corticosteroid therapies (topical, inhaled, or systemic).

### 2.3. International Personality Item Pool 50-Item Questionnaire (IPIP-50)

The International Personality Item Pool 50-item questionnaire (IPIP-50) was used to assess parental personality traits. The IPIP-50 is a public-domain, abbreviated measure of the FFM comprising 50 statements (10 items per trait) that respondents rate on a 5-point Likert scale (1 = “very inaccurate” to 5 = “very accurate”) [15,16]. Negative items were reversely scored after inspection of item wording and corrected item–total correlations; subscale scores were then obtained by summing the relevant items (possible range per trait: 10–50), with higher scores indicating greater expression of the trait. The instrument has been adapted and validated in Croatian samples. Internal consistency was evaluated using Cronbach’s *α*: Extraversion *α* = 0.83, Agreeableness *α* = 0.79, Conscientiousness *α* = 0.84, Emotional stability *α* = 0.86, and Intellect *α* = 0.64, indicating acceptable-to-good reliability for most subscales.

### 2.4. 8-Item Morisky Medication Adherence Scale (MMAS-8)

The 8-item Morisky Medication Adherence Scale (MMAS-8) was used to assess parental self-reported adherence to their child’s prescribed topical/supportive regimen. Permission to use the MMAS-8 was obtained from the instrument’s copyright holder, after which the scale was adapted, translated into Croatian, and psychometrically validated, showing internal consistency comparable to that reported in prior MMAS-8 studies [26,27]. The MMAS-8 comprises seven dichotomous items and one 5-point item; responses were scored according to the original MMAS algorithm to produce a total score ranging from 0 to 8, with higher scores indicating better adherence. For descriptive reporting, we applied conventional cut-offs (low adherence < 6; medium adherence 6 to <8; high adherence = 8), while the MMAS-8 total score was analyzed as a continuous variable in correlational and multivariable analyses.

### 2.5. Statistical Analysis

Statistical analyses were performed in JASP (v.0.18.1.0). Normality of continuous variables was assessed using the Kolmogorov–Smirnov test. Continuous variables are presented as mean ± standard deviation (SD) and categorical variables are reported as frequencies and percentages. Internal consistency of multi-item scales (IPIP subscales and MMAS-8) was evaluated with Cronbach’s alpha (95% CI). Associations between personality subscale scores and adherence (MMAS-8 total score) were examined with Spearman’s rank correlation coefficients. For comparisons of non-normally distributed continuous variables between two or more groups, Mann–Whitney U and Kruskal–Wallis tests were used, respectively. Two-sided *p*-values < 0.05 were considered statistically significant.

## 3. Results

### 3.1. Descriptive Characteristics of Participants

A total of 90 parents participated in the study. The median age of participants was 35 years (IQR 32–37); the sample was predominantly female (77/90, 85.6%) and had a median of 2 children (IQR 1–2). Most participants reported a university degree or higher education (59/90, 65.5%), were employed (79/90, 87.8%), and the majority were not healthcare workers (73/90, 81.1%). Systemic comorbidities were reported by 8 participants (8.9%), and a personal or family history of atopy was present in 56 participants (62.2%). With respect to parents’ own corticosteroid history, 47 (52.2%) reported prior topical corticosteroid use, 18 (20.0%) reported prior inhaled corticosteroid use, and 18 (20.0%) reported prior systemic corticosteroid use (Table 1).

### 3.2. Results of the 8-Item Morisky Medication Adherence Scale

Adherence measured by MMAS-8 had a mean of 5.99 (SD 1.61) and a median of 6.00. The Croatian MMAS-8 showed good reliability, comparable to that reported in validation studies of the MMAS-8 in diverse populations (Cronbach’s *α* = 0.56; 95% CI 0.36–0.75) [26,27]. Using conventional MMAS-8 cut-offs, 36 parents (40.0%) were classified as having low adherence (<6), 41 (45.6%) as medium adherence (6 to <8), and only 13 parents (14.4%) reported high adherence (=8) (Table 2).

### 3.3. Results of the International Personality Item Pool 50-Item Questionnaire

In our sample, parents tended to score relatively high on traits related to social cooperativeness and dutifulness, with conscientiousness showing the highest mean level (mean item score 4.00) and agreeableness also elevated (mean 3.94). Extraversion and intellect showed moderate mean scores (3.42 and 3.50, respectively), while emotional stability was somewhat lower on average (3.21). Reliability analyses indicated acceptable-to-good internal consistency for four of the five scales (*α* = 0.83–0.86 for extraversion, agreeableness, conscientiousness, and emotional stability); the intellect scale displayed lower reliability (*α* = 0.64), which should be taken into account when interpreting findings for that dimension (Table 3).

### 3.4. Associations Between Personality Traits and Adherence

The only personality traits significantly associated with adherence were conscientiousness and emotional stability, where more conscientious and more emotionally stable participants reported higher scores (Table 4).

### 3.5. Associations Between Participant Characteristics and Adherence

Among the examined characteristics, only parent age and systemic corticosteroid use showed significant associations with adherence. Older age was linked to lower adherence (Spearman’s *ρ* = −0.266, *p* = 0.011), and systemic corticosteroid use was also negatively associated (*ρ* = −0.290, *p* = 0.006). This indicates that younger participants and those not using systemic corticosteroids were more likely to adhere (Table 5).

## 4. Discussion

AD in childhood imposes a persistent burden on families, as optimal management ordinarily requires consistent emollient therapy and intermittent, well-timed application of topical anti-inflammatory medications [5]. Parents’ daily implementation of these regimens therefore critically determines treatment effectiveness; nevertheless, caregiver adherence is often inadequate in numerous contexts [29].

In this study, we investigated the relationship between self-reported adherence to children’s AD therapy (MMAS-8) and stable parental dispositional qualities, as assessed by the IPIP-50. The clearest result was that better adherence was moderately and robustly correlated with higher parental conscientiousness, whereas a smaller but significant positive correlation was found with higher emotional stability (lower neuroticism); extraversion, agreeableness, and intelligence were not associated with adherence in our sample. These findings are broadly consonant with prior research showing that dispositional traits, and conscientiousness in particular, predict health behaviors and medication adherence across different medical conditions [30,31].

What could explain why conscientiousness has the strongest connection? Conscientiousness encompasses organized, deliberate, dutiful, and self-disciplined tendencies that seamlessly translate into the formation of routines, prospective memory, and persistence essential for consistent topical care (e.g., daily emollient application and scheduled topical corticosteroid use during flare-ups) [31,32]. Meta-analytic work indicates that conscientiousness is among the most reliable personality predictors of adherence and other preventive health behaviors, consistent with our moderate Spearman *ρ* ≈ 0.43 finding [30].

Practically, conscientious caregivers are more likely to form implementation intentions, adopt reminders, and incorporate treatments into habitual family routines-low-cost strategies that can be actively promoted in clinics [33]. Evidence for implementation intentions improving health behavior supports this approach [22].

Emotional stability (the inverse of neuroticism) showed a weaker but meaningful effect. Low emotional stability correlates with increased worry, emotional reactivity, and a tendency to catastrophize ambiguous bodily symptoms [34]. In the context of AD, this may exacerbate corticophobia (fear of topical corticosteroids) or lead to inconsistent application patterns influenced by transient anxiety, consequently diminishing consistent adherence. Several studies and reviews underscore how corticosteroid fears and misunderstanding of risk are significant adherence barriers in pediatric AD. Our observed link between emotional stability and adherence aligns with these cognitive–affective mechanisms [9,29,35]. These findings also suggest that psychological therapies aimed at parental anxiety and emotional regulation may enhance treatment adherence in pediatric AD. Parents with lower emotional stability may benefit from structured psychological support aimed at reducing anxiety, improving coping strategies, and enhancing emotional regulation. Such interventions have been associated with improved adherence and disease management in other chronic pediatric conditions and represent a promising complementary approach alongside standard educational programs [20]. Although intervention effects were not examined in the present study, this represents an important direction for future research.

Topical corticosteroid phobia deserves explicit emphasis since it is a frequently documented reason caregivers stray from prescribed regimens. Validated tools (e.g., TOPICOP) measure corticophobia and have demonstrated that safety concerns, prior adverse experiences, or inconsistent communication from clinicians lead to underutilization or inappropriate self-modification of therapy [9]. In this context, topical corticosteroid phobia may plausibly mediate the relationship between parental personality traits and treatment adherence. Parents with lower emotional stability may be particularly susceptible to anxiety-driven concerns about topical corticosteroids, resulting in avoidance or inconsistent use. Although corticophobia was not directly evaluated in the present study, future research incorporating validated instruments such as the TOPICOP questionnaire is warranted to formally test this mediating pathway.

Measurement issues merit discussion. MMAS-8 is extensively utilized and possesses established predictive validity for medication adherence across diverse clinical settings; nonetheless, its psychometric efficacy varies according to language and demographic group [36]. In our Croatian sample, the MMAS-8 showed internal consistency values similar to those reported in previous MMAS-8 studies (*α* = 0.56), reflecting known psychometric characteristics of brief self-report adherence measures. This consideration underscores the necessity of complementing self-report with objective adherence methods (such as electronic monitoring, prescription refill metrics, and weighing of topical tubes) whenever feasible. For instance, electronic monitoring often shows lesser adherence than self-reporting and gives detailed information on when and how often something was used [37].

From a clinical translation perspective, the data indicate viable and scalable interventions. For caregivers with poor conscientiousness, behavioral implementation aids, such as explicit if–then plans (implementation intentions), smartphone reminders, or habit-linking tactics (applying emollient after teeth brushing), are cost-effective and evidence-based methods to enhance routine enactment [38,39,40]. Meta-analyses and systematic reviews indicate that reminder systems (SMS, apps) and simple behavioral prompts improve medication adherence across disease areas, and several digital interventions have been piloted successfully in dermatology. Embedding such supports within existing Atopy School programs could be efficient and acceptable [5].

For caregivers exhibiting elevated trait anxiety (lower emotional stability), interventions that specifically address disease beliefs and corticophobia may be preferable. Educational modules that provide clear, dose-specific safety information about topical corticosteroids and explicitly address common myths reduce fear and increase confidence. When these are combined with short cognitive–behavioral therapy to change catastrophic thinking, they can help with the cognitive-affective routes that undermine adherence [40,41,42].

Atopy School models, which are structured education programs for caregivers, represent a natural delivery platform for personality-informed adherence modules. Evaluations of Atopy Schools demonstrate improved caregiver knowledge, confidence, and satisfaction, and establish such programs as successful environments for implementing specialized behavioral and educational supports. Nevertheless, the content and level of intensity vary greatly, and more standardization and evidence-based improvement are required [5,43].

Pragmatically, the evidence base supports a dual approach for translation: cognitive-targeted education to reduce corticophobia and correct misconceptions [20], alongside simple behavioral strategies (implementation intentions, reminders, digital prompts) to facilitate routine enactment for less conscientious caregivers [22,44]. Meta-analyses and systematic reviews demonstrate that multi-component interventions and mHealth reminders can produce modest yet clinically significant improvements in adherence across chronic diseases [20,45]. Furthermore, objective adherence monitoring (electronic devices, refill data) both clarifies baseline non-adherence and enables prompt, personalized follow-up [45,46].

The study has several strengths that enhance confidence in the findings. We used a validated, public-domain personality measure (IPIP-50), which has documented validity in Croatian samples, and assessed internal consistency within our cohort. Recruiting parents within a real-world educational clinic setting improves ecological validity and demonstrates the feasibility of brief psychosocial measurement in routine dermatology practice [16].

Our findings have direct practical implications: brief personality screening might be incorporated into Atopy School intake procedures to identify caregivers requiring extra adherence support. Low-burden tools combined with nurse-led counseling and automated reminders can be piloted rapidly. Embedding such stratified care pathways may increase efficiency and target limited resources to families most likely to benefit across settings.

Beyond personality traits, adherence behavior is shaped by a broader psychosocial setting. Factors such as parental educational level, health literacy, perceived social support, and prior experience with dermatological treatment may moderate the influence of personality on adherence. For example, higher educational attainment or stronger social support networks may buffer the negative impact of lower conscientiousness or higher anxiety on treatment implementation, whereas limited health literacy may amplify trait-related vulnerabilities. Although these variables were not examined as moderators in the current study, they represent important targets for future investigations seeking to develop more comprehensive adherence models in pediatric AD.

Clinical characteristics of the child’s disease may also influence the relationship between parental personality traits and adherence. Disease severity and symptom burden could act as contextual moderators, potentially strengthening or attenuating trait–adherence associations. More severe or persistent symptoms may motivate stricter adherence in some caregivers, while in others, they may increase distress, helplessness, or avoidance behaviors. Because objective measures of disease severity (e.g., SCORAD or EASI) were not included in the present study, we were unable to evaluate this hypothesis directly. Future studies incorporating standardized severity assessments are needed to clarify how clinical context interacts with parental dispositional factors.

Because our data are cross-sectional, causal relationships cannot be inferred. While stable parental dispositional traits may plausibly influence treatment adherence, the reverse is also possible, as chronic non-adherence, repeated treatment failures, or the ongoing stress of managing a child’s uncontrolled AD may affect caregivers’ mood and could even produce state-like shifts on personality measures [47,48,49]. Longitudinal or experimental studies are therefore required to establish temporal order and clarify causal pathways, as well as to examine whether the observed associations between conscientiousness, emotional stability, and adherence remain stable over time or change with illness duration and accumulated caregiving experience.

Reliance on self-report adherence (MMAS-8) introduces social-desirability and recall biases. As noted earlier, the scale showed limited internal consistency in this sample, so triangulation with objective adherence measures would strengthen inference in future studies. Single-center recruitment (Atopy School attendees) may limit generalizability to less engaged populations encountered in primary care. Sample size, adequate for detecting moderate correlations, may nonetheless have limited power to detect small effects or interactions (for example, trait × socioeconomic status). Finally, residual confounding by unmeasured variables (health literacy, detailed illness perceptions, and family functioning) remains possible; future studies should integrate validated measures of illness perception and corticophobia (e.g., B-IPQ, TOPICOP) to test mediation hypotheses.

## 5. Conclusions

Within our sample, parental conscientiousness, and to a smaller extent emotional stability, correlated with higher self-reported adherence to children’s AD regimens. These findings support the inclusion of dispositional factors in adherence models and point to pragmatic, personality-sensitive strategies that could be embedded within multidisciplinary atopy care (education programs, digital reminders, and brief cognitive–behavioral interventions). Addressing both behavioral implementation barriers and explicit cognitive concerns, such as corticophobia, may improve regimen implementation and, ultimately, child outcomes in pediatric AD.

## Figures and Tables

**Table 1:** Descriptive characteristics of the parents of pediatric patients with AD (n = 90).

Variable	Levels	Value
Age		35 (IQR 32–27)
Sex		
	Male	13 (14.4%)
	Female	77 (85.6%)
Number of children		2 (IQR 1–2)
Marital status		
	Married	81 (90.0%)
	Extramarital community	6 (6.7%)
	Never married	2 (2.2%)
	Widow	1 (1.1%)
Education		
	High school	22 (24.4%)
	College	9 (10.0%)
	University degree	48 (53.3%)
	Doctorate	11 (12.2%)
Employment		
	Unemployed	11 (12.2%)
	Employed	79 (87.8%)
Health worker	Yes No	17 (18.9%) 23 (81.1%)
Systemic comorbidities	Yes No	8 (8.9%) 92 (91.1%)
Atopia history	Yes No	56 (62.2%) 44 (37.8%)
TCS * use	Yes No	47 (52.2%) 53 (47.8%)
ICS ** use	Yes No	18 (20.0%) 82 (80%)
OCS *** use	Yes No	18 (20.0%) 82 (80%)

* TCS—topical corticosteroid, ** ICS—inhaled corticosteroid, *** OCS—oral corticosteroid.

**Table 2:** Distribution of adherence (MMAS-8) (n = 90).

MMAS-8 Adherence Category ^1^	Value
Low adherence (<6)	36 (40.0%)
Medium adherence (6 to <8)	41 (45.6%)
High adherence (=8)	13 (14.4)

^1^ MMAS-8 total score-mean (SD) = 5.99 (1.61). Internal consistency: Cronbach’s *α* = 0.56 (95% CI 0.36–0.75).

**Table 3:** IPIP-50 subscale means and internal consistency (n = 90).

Scale	Mean (SD) (1–5)	Cronbach’s *α* (95% CI)
Extraversion	3.42 (0.59)	0.83 (0.82–0.83)
Agreeableness	3.94 (0.53)	0.79 (0.78–0.79)
Conscientiousness	4.00 (0.55)	0.84 (0.83–0.84)
Emotional stability	3.21 (0.67)	0.86 (0.85–0.86)
Intellect	3.50 (0.42)	0.64 (0.63–0.64)

**Table 4:** Spearman correlations between personality traits and adherence score (n = 90).

Variable	MMAS	Extraversion	Agreeableness	Conscientiousness	Emotional Stability	Intellect
1.	1.00					
2.	−0.02	1.00				
3.	0.06	0.25 *	1.00			
4.	0.43 **	−0.08	0.16	1.00		
5.	0.22 *	0.15	0.09	0.06	1.00	
6.	0.13	0.25 *	0.22 *	0.09	0.03	1.00

* *p* < 0.05, ** *p* < 0.001.

**Table 5:** Spearman correlations between participant characteristics and adherence score (n = 90).

Variable	Estimate (Spearman’s *ρ*)	*p*-Value
Age (years)	−0.266	0.011
Sex (female)	−0.175	0.099
Number of children	−0.076	0.477
Marital status (married/cohabiting)	0.199	0.060
Education level (higher education)	0.045	0.671
Employment (employed)	−0.047	0.660
Health worker (yes)	−0.008	0.938
Systemic comorbidities (yes)	−0.079	0.458
Atopy history (yes)	−0.087	0.414
TCS * use (yes)	−0.128	0.228
ICS ** use (yes)	−0.031	0.773
OCS *** use (yes)	−0.290	0.006

* TCS—topical corticosteroid, ** ICS—inhaled corticosteroid, *** OCS—oral corticosteroid.

## Data Availability

The original contributions presented in this study are included in the article. Further inquiries can be directed to the corresponding author.
