# Correction: Markota Čagalj et al. Personality Traits and Treatment Adherence Among Parents of Children with Atopic Dermatitis. *Medicina* 2026, *62*, 59

**DOI:** 10.3390/medicina62112067

**Published:** 2026-10-26

**Authors:** Adela Markota Čagalj, Zdenka Šitum Čeprnja, Dina Lešin Gaćina, Jasna Petrić Duvnjak, Maja Pavić, Tina Gogić Salapić, Bepa Pavlić, Shelly Melissa Pranić, Dubravka Vuković

**Affiliations:** 1 Department of Dermatology and Venereology, University Hospital of Split, Spinčićeva 1, 21000 Split, Croatia; adela.markota@gmail.com (A.M.Č.); bepa.pavlic55@gmail.com (B.P.); 2 Department of Ophthalmology, Zagreb University Hospital Center, Kišpatićeva 12, 10000 Zagreb, Croatia; 3 Poliklinika Pediatri, Kranjčevićeva 45, 21000 Split, Croatia; 4 Department of Health Studies, University of Split, Ulica Ruđera Boškovića 35, 21000 Split, Croatia; 5 Department of Public Health, School of Medicine, University of Split, Šoltanska 2, 21000 Split, Croatia; 6 Cohrane Croatia, Šoltanska 2, 21000 Split, Croatia; 7 School of Medicine, University of Split, Šoltanska 2, 21000 Split, Croatia

## Missing Copyright

In the original article [1], the Morisky Medication Adherence Scale-8 (MMAS-8) was used, and a license was obtained from MMAR, LLC DBA Adherence, but the copyright statement was not included.

## Back Matter Correction

New Acknowledgements have been added to state the copyright; the authors requested for this to be updated as below:

**Acknowledgments:** Use of the ©MMAS-8 is protected by US copyright and trademark laws. Permission for use of the scale and its coding is required. A license agreement is available from MMAR, LLC, www.moriskyscale.com (accessed on 21 December 2025).

The authors state that the scientific conclusions are unaffected. This correction was approved by the Academic Editor. The original publication has also been updated.
